# TPH2 Deficiency Influences Neuroplastic Mechanisms and Alters the Response to an Acute Stress in a Sex Specific Manner

**DOI:** 10.3389/fnmol.2018.00389

**Published:** 2018-10-30

**Authors:** Paola Brivio, Giulia Sbrini, Polina Peeva, Mihail Todiras, Michael Bader, Natalia Alenina, Francesca Calabrese

**Affiliations:** ^1^Department of Pharmacological and Biomolecular Sciences, University of Milan, Milan, Italy; ^2^Cardiovascular and Metabolic Diseases, Max-Delbrück-Center for Molecular Medicine (MDC), Berlin, Germany; ^3^Charite-University Medicine, Berlin, Germany; ^4^Institute of Translational Biomedicine, St. Petersburg State University, St. Petersburg, Russia

**Keywords:** serotonin, TPH2, Bdnf, neuroplasticity, stress, immediate early gene

## Abstract

Dysregulations of the central serotoninergic system have been implicated in several psychopathologies, characterized by different susceptibility between males and females. We took advantage of tryptophan hydroxylase 2 (TPH2) deficient rats, lacking serotonin specifically in the brain, to investigate whether a vulnerable genotype can be associated with alterations of neuronal plasticity from the early stage of maturation of the brain until adulthood. We found a significant increase, in both gene and protein expression, of the neurotrophin brain-derived neurotrophic factor (BDNF), in the prefrontal cortex (PFC) of adult TPH2-deficient (TPH2^−/−^) male and female rats in comparison to wild type (TPH2^+/+^) counterparts. Interestingly, a development-specific pattern was observed during early postnatal life: whereas the increase in *Bdnf* expression, mainly driven by the modulation of *Bdnf* isoform IV was clearly visible after weaning at postnatal day (pnd) 30 in both sexes of TPH2^−/−^ in comparison to TPH2^+/+^ rats, at early stages (pnd1 and pnd10) *Bdnf* expression levels did not differ between the genotypes, or even were downregulated in male TPH2^−/−^ animals at pnd10. Moreover, to establish if hyposerotonergia may influence the response to a challenging situation, we exposed adult rats to an acute stress. Although the pattern of corticosterone release was similar between the genotypes, neuronal activation in response to stress, quantified by the expression of the immediate early genes activity regulated cytoskeleton associated protein (*Arc*) and Fos Proto-Oncogene (*cFos*), was blunted in both sexes of animals lacking brain serotonin. Interestingly, although upregulation of *Bdnf* mRNA levels after stress was observed in both genotypes, it was less pronounced in TPH2^−/−^ in comparison to TPH2^+/+^ rats. In summary, our results demonstrated that serotonin deficiency affects neuroplastic mechanisms following a specific temporal pattern and influences the response to an acute stress.

## Introduction

Serotonin 5-hydroxytryptamine (5-HT) was originally discovered as cardiovascular hormone but has later gained prominence as neurotransmitter or neuromodulator widely distributed in the central nervous system (CNS) and peripheral nervous systems. Nevertheless, by far the highest amounts of the monoamine are produced in the intestine and released into the blood stream, where it is taken up by platelets which transport it through the circulation and release it at sites of their activation.

5-HT biosynthesis occurs from the essential aminoacid tryptophan in two steps, and tryptophan hydroxylase 2 (TPH2) is the rate-limiting enzyme in this process. As it has been discovered in 2003 (Walther et al., 2003), 5-HT is produced by two different enzymes, TPH1 and TPH2, in the gut and in the brain, respectively. Since in adulthood it cannot pass the blood-brain barrier, these two enzymes define two 5-HT systems with independent regulation and different function. Although in the adult brain 5-HT is produced only by serotonergic neurons in raphe nuclei, during development there are other sources of this monoamine, including maternal 5-HT, that is actively transported through the placental brush border cells via the serotonin transporter (SERT; Cool et al., 1990; Carrasco et al., 2000; Kliman et al., 2018). Moreover, until postnatal day (pnd) 12, the blood-brain barrier is immature (Ribatti et al., 2006) and 5-HT, produced by TPH1 starting at embryonic day 14 in rodents (Côté et al., 2007) can reach the brain. In the early phases of embryonic and postnatal life, 5-HT is a trophic factor that modulates not only cell proliferation, migration and differentiation in the brain and in peripheral tissues (Azmitia, 2001; Buznikov et al., 2001; Gaspar et al., 2003) but also cell survival and synaptogenesis, through its role in the connective organization of the CNS (Gaspar et al., 2003).

Altered 5-HT concentration in the CNS have been implicated in several psychopathologies (H. M., 1994; Brewerton, 1995; Heninger, 1995), characterized by a different susceptibility between males and females such as psychiatric disorders (Breslau et al., 1997; Young and Korszun, 2010), as well as in an altered antidepressant response and tolerance among males and females (Kornstein et al., 2000). Moreover, serotonergic pathways, serotonin synthesis (Nishizawa et al., 1997), the levels of 5-HT metabolites (Gottfries et al., 1974), as well as the expression and activity of different receptors and transporters (Jovanovic et al., 2008) are modulated by sex. In this work we investigate the impact of sex on susceptibility to alterations in the serotonergic system using a new animal model deficient in brain serotonin synthesis, TPH2-deficient (TPH2^−/−^) rats (Kaplan et al., 2016).

Hence, we used male and female TPH2^−/−^ rats to investigate the influence of brain serotonin depletion on neuronal plasticity, in particular on the brain-derived neurotrophic factor (BDNF) from early life until adulthood. Indeed, it is well known that there is a correlation between 5-HT and BDNF (Martinowich and Lu, 2008), the main players in modulation of neurogenesis and neuroplastic mechanisms across lifespan (Homberg et al., 2014). Accordingly, we previously demonstrated that SERT knockout rats showed impaired neuroplasticity starting from the third week of life until adulthood (Calabrese et al., 2013). Moreover, in order to evaluate if hyposerotonergia may alter the response to a challenging condition, we exposed adult TPH2^−/−^ and wild type (TPH2^+/+^) rats to an acute stress focusing on BDNF modulations, since it is known that there is a strong relationship between acute stress response and neuroplasticity (Calabrese et al., 2009) and that 5-HT may be involved in the regulation of *Bdnf* expression during challenges (Foltran and Diaz, 2016). All the molecular analyses were conducted in the prefrontal cortex (PFC), a brain area involved in modulation of mood disorders and strictly connected not only with the neuromodulatory serotonin actions but also with BDNF (Duman and Monteggia, 2006).

## Materials and Methods

### Animals

Animals were kept under standardized conditions with an artificial 12/12-h light/dark cycle (lights on at 6 a.m.), room temperature of 22°C, and approximately 80% humidity with access to food (Ssniff, Soest, Germany) and water *ad libitum*. Male and female TPH2-deficient (TPH2^−/−^) and wild type (TPH2^+/+^) rats on the dark agouti background (Kaplan et al., 2016) were used in the experiments. A cohort of adult (12–14 weeks of age) TPH2^+/+^ and TPH2^−/−^ male and female animals (6–14 animals for each experimental group) was used for the basal measurements. A second cohort of adult male and female TPH2^+/+^ and TPH2^−/−^ rats (4–7 per sex per condition, see below “Stress Procedure” section) was used for the stress experiment. For the developmental analysis male and female rats were sacrificed at pnd 1, 10 and 30 (5–10 animals per age, sex and genotype). All procedures were approved by the ethical committee of the local government (LAGeSo, Berlin, Germany).

### Stress Procedure

Adult male and female TPH2^+/+^ and TPH2^−/−^ rats were exposed to acute restraint stress: rats were placed in an air-accessible apparatus for 1 h. The size of the container was similar to the size of the animal, which made the animal almost immobile in it. Half of the stressed animals were sacrificed immediately at the end of the stress session (stress0’), while the other groups were sacrificed 60 min after the end of the stress (stress60’). Unstressed animals served as control group (baseline, BL). Animals were sacrificed by decapitation and trunk blood was collected for the analysis of plasma corticosterone levels.

### Brain Tissue Collection

Animals were sacrificed by decapitation and brain regions of interest were rapidly dissected, frozen on dry ice and stored at −80°C for the molecular analyses. Dissections were performed according to the atlas of Paxinos and Watson (1998). In detail, frontal lobe for pnd1-10-30 rats and PF for adult rats were dissected from 2-mm-thick slices (PF defined as Cg1, Cg3 and IL subregions corresponding to plates 6–9 (approximate weight 8 mg)). The left hemisphere was taken for protein whereas the right was taken for RNA.

### RNA Preparation and Gene Expression Analysis by Quantitative Real-Time PCR

Total RNA was isolated by a single step of guanidinium isothiocyanate/phenol extraction using PureZol RNA isolation reagent (Bio-Rad Laboratories, Italy) according to the manufacturer’s instructions and quantified by spectrophotometric analysis. Following total RNA extraction, the samples were processed for real-time polymerase chain reaction (RT-PCR) to assess total *Bdnf*, long 3′-UTR *Bdnf* and the exons IV and VI, activity regulated cytoskeleton associated protein (*Arc*) and Fos Proto-Oncogene (*cFOS*) mRNA levels. An aliquot of each sample was treated with DNAse (Life Technologies, Italy) to avoid DNA contamination. RNA was analyzed by TaqMan qRT-PCR instrument (CFX384 real time system, Bio-Rad Laboratories, Italy) using the iScript™ one-step RT-PCR kit for probes (Bio-Rad Laboratories, Italy). Samples were run in 384 well formats in triplicate as multiplexed reactions with a normalizing internal control (36B4). Primers and probes (Tables 1A,B) were purchased from Eurofins MWG-Operon (Germany) and Life Technologies (Italy). Thermal cycling was initiated with an incubation at 50°C for 10 min (RNA reverse transcription) and then at 95°C for 5 min (TaqMan polymerase activation). After this initial step, 39 cycles of PCR were performed. Each PCR cycle consisted of heating the samples at 95°C for 10 s to enable the melting process and then for 30 s at 60°C for the annealing and extension reactions. A comparative cycle threshold method was used to calculate the relative target gene expression (Livak and Schmittgen, 2001).

**Table 1A:** Sequences of forward and reverse primers and probes used in real-time polymerase chain reaction (RT-PCR) analysis and purchased from Eurofins MWG-Operon (Germany; **A**) and from Life Technologies, which did not disclose the sequences **(B)**. **(A)** Forward and reverse primers and probes purchased from Eurofins MWG-Operon (Germany).

Gene	Forward primer	Reverse primer	Probe
Arc	GGTGGGTGGCTCTGAAGAAT	ACTCCACCCAGTTCTTCACC	GATCCAGAACCACATGAATGGG
cFos	TCCTTACGGACTCCCCAC	CTCCGTTTCTCTTCCTCTTCAG	TGCTCTACTTTGCCCCTTCTGCC
Total Bdnf	AAGTCTGCATTACATTCCTCGA	GTTTTCTGGAGGGACAGTTTAT	TGTGGTTTGTTGCCGTTGCCAAG
36b4	TTCCCACTGGCTGAGGT	CGCAGCCGCTGC	AAGGCCTTCCTGGCCGATCCATC

**Table 1B:** **(B)** Forward and reverse primers and probes purchased from Life Technologies.

Gene	Accession number	Assay ID
Bdnf Long 3’UTR	EF125675	Rn02531967_s1
Bdnf isoform IV	EF125679	Rn01484927_m1
Bdnf isoform VI	EF125680	Rn01484928_m1

### Protein Extraction and Western Blot Analysis

Western blot analysis was used to investigate mature BDNF in the crude synaptosomal fraction. Tissues were manually homogenized using a glass-glass potter in a pH 7.4 cold buffer containing 0.32 M sucrose, 0.1 mM EGTA, 1 mM HEPES solution in the presence of a complete set of protease (Roche) and phosphatase (Sigma-Aldrich) inhibitors. The total homogenate was centrifuged at 2,500 rpm for 10 min at 4°C. The supernatant obtained was further centrifuged at 10,000 *g* for 15 min at 4°C to obtain the pellet corresponding to the crude synaptosomal fraction which was re-suspended in a buffer (20 mM HEPES, 0.1 mM dithiothreitol, 0.1 mM EGTA) with protease and phosphatase inhibitors. Total protein content was measured according to the Bradford Protein Assay procedure (Bio-Rad Laboratories), using bovine serum albumin as a calibration standard. Equal amounts of protein were run under reducing conditions on the criterion TGX precast gels (Bio-Rad Laboratories) and then electrophoretically transferred onto nitrocellulose membranes (Bio-Rad Laboratories). The blots were blocked with 10% nonfat dry milk and then incubated with the primary antibodies (mature BDNF: 1:1,000 (Icosagen), 4° O/N; β-actin 1:10,000 (Sigma), 4°C, O/N). Membranes were then incubated for 1 h at room temperature with the appropriate secondary antibody (mature BDNF: anti-mouse, 1:1,000, RT, 1 h; β-actin: anti-mouse1:10,000). Immunocomplexes were visualized by chemiluminescence using the Western Lightning Clarity ECL (Bio-Rad Laboratories) and the Chemidoc MP imaging system (Bio-Rad Laboratories). BDNF expression was quantified by the evaluation band densities, normalized to the β-actin (ImageLab, Bio-Rad Laboratories).

### Analysis of Plasma Corticosterone Levels

Samples of blood from each rat were collected in MiniCollect K3EDTA (Greiner Bio-One GmbH, Frickenhausen, Deutschland) tubes. Plasma was separated by centrifugation for 10 min at 1,300 *g*, 4° and corticosterone was determined by an enzyme-linked immunosorbent assay (ELISA) using a commercial kit (IBL international, Hamburg, Germany) according to the manufacturers’ instructions.

### Statistical Analyses

All the statistical analyses were conducted by using “IBM SPSS Statistics, version 24.” The effects of genotype and sex were analyzed with the two-way analysis of variance (ANOVA) with genotype and sex as independent factors. When appropriate, differences between the individual groups were analyzed by Fisher’s protected least significant difference (PLSD). The effects of stress, genotype and sex in the acute stress experiments were analyzed with three-way ANOVA with Fisher’s PLSD. Significance for all tests was assumed for *p* < 0.05. Data are presented as means ± standard error mean (SEM). For graphic clarity, all qPCR and western blot results are normalized to male TPH2^+/+^ values that are taken as 100%.

## Results

### Analysis of *Bdnf* Transcripts and BDNF Protein Levels in the PFC of TPH2^−/−^ Male and Female Adult Rats

**Figure 1:**
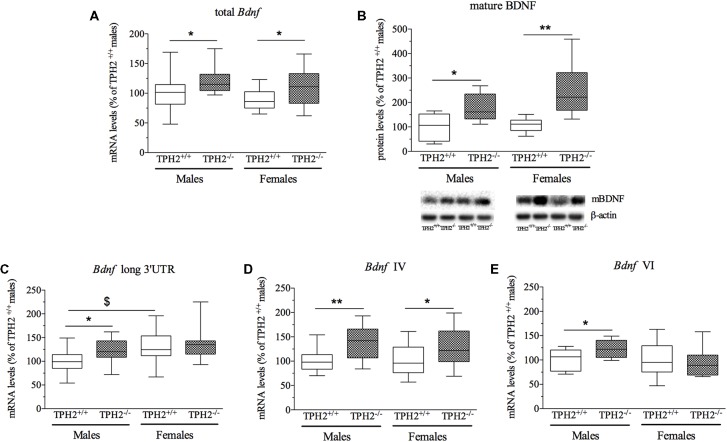
Analysis of brain-derived neurotrophic factor (*Bdnf*) transcripts mRNA levels **(A,C,D,E)** and mature BDNF protein levels **(B)** in the crude synaptosomal fraction of prefrontal cortex (PFC) of tryptophan hydroxylase 2 (TPH2^+/+^) and TPH2^−/−^ adult male and female rats. The data, expressed as % of TPH2^+/+^ male rats, represent the mean ± standard error mean (SEM) of at least 14 independent determinations for the RNA and six for the western blot. *p < 0.05, **p < 0.01 vs. TPH2^+/+^ of the same sex; ^$^*p* < 0.05 vs. TPH2^+/+^/male (two-way analysis of variance (ANOVA) with Fisher’s protected least significant difference (PLSD).

We first investigated whether the mRNA expression of total *Bdnf* was modulated by the lack of serotonin, in the PF of both male and female adult rats. As shown in Figure 1A, we found a significant effect of the genotype (*F*_(1–52)_ = 10.232, *p* < 0.01) but not of the sex and interaction genotype × sex. *Post hoc* analysis revealed an increase of total *Bdnf* mRNA levels both in TPH2^−/−^ male rats (+23%, *p* < 0.05 vs. TPH2^+/+^) and in TPH2^−/−^ female rats (+25%, *p* < 0.05 vs. TPH2^+/+^). However, no difference was found between the sexes for both genotypes.

To assess if the changes in total *Bdnf* mRNA were paralleled by alterations in BDNF protein, we investigated the levels of the mature form of BDNF (mBDNF) in crude synaptosomal fraction (Figure 1B). Two-way ANOVA showed a significant effect of the genotype (*F*_(1–27)_ = 17.673, *p* < 0.001) with no genotype × sex interaction. As revealed by *post hoc* analysis, the levels of mBDNF were significantly increased in both male (+78%, *p* < 0.05 vs. TPH2^+/+^/male) and female (+139%, *p* < 0.01 vs. TPH2^+/+^/female) TPH2^−/−^ rats in comparison to the TPH2^+/+^ counterparts (Figure 1B).

Based on the enhancement of total *Bdnf* levels found in TPH2^−/−^ rats, we decided to evaluate if serotonin-deficiency in the CNS differently affects the expression of major *Bdnf* transcripts. In particular, we quantified the expression levels of long 3’UTR *Bdnf* transcripts, and isoforms IV and VI to establish their contribution to the observed modulation in total *Bdnf*.

*Bdnf* long 3’UTR was significantly modulated by the genotype (*F*_(1–53)_ = 4.703, *p* < 0.05), with an up-regulation of *Bdnf* long 3’UTR mRNA levels specifically in male TPH2^−/−^ in comparison to TPH2^+/+^ rats (+28%, *p* < 0.05 vs. TPH2^+/+^/male). Moreover, the significant effect of the sex (*F*_(1–53)_ = 5.884, *p* < 0.05) reflected the increased expression of *Bdnf* long 3’UTR in female TPH2^+/+^ in comparison to male counterparts (+30%, *p* < 0.05 vs. TPH2^+/+^/male; Figure 1C). On the contrary, two-way ANOVA did not show a significant genotype × sex interaction.

Similarly to the results obtained for the total form of the neurotrophin, we observed a significant effect of the genotype (*F*_(1–54)_ = 14.017, *p* < 0.05) for *Bdnf* isoform IV (Figure 1D), with no effect of sex and genotype × sex interaction. Accordingly, we found a significant upregulation of *Bdnf* IV expression in TPH2^−/−^ of both sexes (male: +39%, *p* < 0.01 vs. TPH2^+/+^/male; female: +27%, *p* < 0.05 vs. TPH2^+/+^/female).

On the contrary, as shown in Figure 1E, we found a significant genotype × sex interaction (*F*_(1–53)_ = 4.580, *p* < 0.05) for *Bdnf* isoform VI. Indeed, *Bdnf* VI increased only in male TPH2^−/−^ rats (+22%, *p* < 0.05 vs. TPH2^+/+^/male).

### Analysis of *Bdnf* Transcripts in the Frontal Lobe of TPH2^−/−^ Male and Female Rats During Postnatal Development

**Figure 2:**
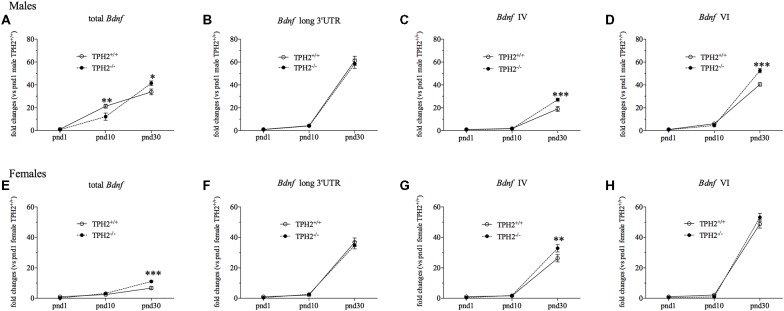
Developmental analysis oftotal *Bdnf* **(A,E)**, *Bdnf* long 3’UTR **(B,F)**, *Bdnf* isoform IV **(C,G)** and *Bdnf* isoform VI **(D,H)** mRNA levels in TPH2^−/−^ male and female rats at postnatal days (pnd) 1, 10 and 30. The data, expressed as fold change to TPH2^+/+^/pnd1, are the mean ± SEM of at least five independent determinations. *p < 0.05, **p < 0.01, ***p < 0.001 vs. TPH2^+/+^ rats/same pnd (two-way ANOVA with Fisher’s PLSD).

To evaluate the impact of serotonin deficiency during development on *Bdnf* expression, we investigated its mRNA levels in TPH2^+/+^ and TPH2^−/−^ rats at different ages of life, namely pnd 1, 10 and 30 (Figure 2).

In male rats, we found a significant effect of the age (*F*_(2–43)_ = 107.444, *p* < 0.001) that is reflected by a significant increase of total *Bdnf* transcripts in both genotypes, at pnd10 compared to pnd1 rats (TPH2^+/+^/pnd10: +1657%, *p* < 0.001 vs. TPH2^+/+^/pnd1; TPH2^−/−^/pnd10: +1467%, *p* < 0.01 vs. TPH2^−/−^/pnd1) but also at pnd30 compared to pnd10 animals (TPH2^+/+^/pnd30: +59%, *p* < 0.001 vs. TPH2^+/+^/pnd10; TPH2^−/−^/pnd30: +240%, *p* < 0.001 vs. TPH2^−/−^/pnd10; Figure 2A). Moreover, we found a significant age × genotype interaction (*F*_(2–43)_ = 7.115, *p* < 0.01). Indeed, the developmental profiles of the *Bdnf* expression differed between the genotypes, with an equal expression at pnd1, decreased total *Bdnf* mRNA levels in TPH2^−/−^ at pnd10 (−43%, *p* < 0.01 vs. TPH2^+/+^/pnd10), and a significant increase at pnd30 (+22%, *p* < 0.05 vs. TPH2^+/+^/ pnd30).

Similarly, we observed a significant effect of the age (*F*_(2–45)_ = 297.514, *p* < 0.001) for *Bdnf* long 3’UTR transcripts with an upregulation from pnd10 to pnd30 in both TPH2^+/+^ (TPH2^+/+^/pnd30: +1315%, *p* < 0.001 vs. TPH2^+/+^/pnd10) and TPH2^−/−^ rats (TPH2^−/−^/pnd30: +1355%, *p* < 0.001 vs. TPH2^−/−^/pnd10; Figure 2B).

Moreover, we found a significant effect of age (*F*_(2–46)_ = 287.421, *p* < 0.001) for *Bdnf* isoform IV, with an increase in both genotypes from pnd10 to pnd30 (TPH2^+/+^/pnd30: +940%, *p* < 0.001 vs. TPH2^+/+^/pnd10; TPH2^−/−^/pnd30: +1587%, *p* < 0.001 vs. TPH2^−/−^/pnd10). Furthermore, there was a significant effect of the genotype (*F*_(1–46)_ = 8.667, *p* < 0.01) and a significant genotype × age interaction (*F*_(2–46)_ = 10.602, *p* < 0.001) with an upregulation of *Bdnf* isoform IV at pnd30 in TPH2^−/−^ rats (+43%, *p* < 0.001 vs. TPH2^+/+^/pnd30; Figure 2C).

Similarly, *Bdnf* isoform VI was significantly modulated by the age (*F*_(2–46)_ = 823.232, *p* < 0.001) with an increase both in TPH2^+/+^ and TPH2^−/−^ from pnd1 to pnd10 (TPH2^+/+^/pnd10: +486, *p* < 0.01 vs. TPH2^+/+^/pnd1; TPH2^−/−^/pnd10: +473, *p* < 0.05 vs. TPH2^−/−^/pnd1) and also from pnd10 to pnd30 (TPH2^+/+^/pnd30: +570%, *p* < 0.001 vs. TPH2^+/+^/pnd10; TPH2^−/−^/pnd30: +1072%, *p* < 0.001 vs. TPH2^−/−^/pnd10). Moreover, we found a significant effect of the genotype (*F*_(1–46)_ = 10.912, *p* < 0.01) and a significant genotype × age interaction (*F*_(2–46)_ = 19.039, *p* < 0.001) with an upregulation of *Bdnf* isoform VI only at pnd30 in TPH2^−/−^ in comparison to TPH2^+/+^ rats of the same age (+30%, *p* < 0.001 vs. TPH2^+/+^/pnd30; Figure 2D).

As shown in Figure 2E, in female rats, we found a significant effect of the age (*F*_(2–41)_ = 105.889, *p* < 0.001) for total *Bdnf* mRNA levels with a significant increase among the three ages. Actually, we observed an enhancement from pnd1 to pnd10 only in TPH2^−/−^ rats (TPH2^−/−^/pnd10: +1624%, *p* < 0.01 vs. TPH2^−/−^/pnd1), while starting from pnd10, an upregulation of total *Bdnf* mRNA levels was seen in both genotypes (TPH2^+/+^/pnd30: +170%, *p* < 0.001 vs. TPH2^+/+^/pnd10; TPH2^−/−^/pnd30: +241%, *p* < 0.001 vs. TPH2^−/−^/pnd10). Moreover, we found a significant increase of total *Bdnf* in TPH2^−/−^ rats compared to TPH2^+/+^ (+64%, *p* < 0.001 vs. TPH2^+/+^/ pnd30) only at pnd30, as supported by the significant effect of the genotype (*F*_(1–41)_ = 8,400, *p* < 0.01) and of the interaction age × genotype (*F*_(2–41)_ = 9.843, *p* < 0.001).

With respect to *Bdnf* long 3’UTR (Figure 2F), we observed a significant effect of the age (*F*_(2–43)_ = 292.426, *p* < 0.001) with a robust upregulation at pnd30 compared to pnd10 in both TPH2^+/+^ (TPH2^+/+^/pnd30: +1601%, *p* < 0.001 vs. TPH2^+/+^/pnd10) and TPH2^−/−^ (TPH2^−/−^/pnd30: +1210%, *p* < 0.001 vs. TPH2^−/−^/pnd10), but we didn’t find any significant interaction of age × genotype.

Similarly, *Bdnf* isoform IV was significantly modulated by the age (*F*_(2–43)_ = 217.038, *p* < 0.001), but not by the genotype in young female rats. Accordingly, we observed an increase in both genotypes from pnd10 to pnd30 (TPH2^+/+^/pnd30: +1484%, *p* < 0.001 vs. TPH2^+/+^/pnd10; TPH2^−/−^/pnd30: +1676%, *p* < 0.001 vs. TPH2^−/−^/pnd10). Furthermore, the lack of serotonin at pnd30 induced an upregulation of *Bdnf* isoform IV in comparison to TPH2^+/+^/pnd30 (+25%, *p* < 0.01 vs. TPH2^+/+^/pnd30; Figure 2G).

In line with what was observed for *Bdnf* long 3’UTR transcripts, *Bdnf* isoform VI was significantly modulated by the age (*F*_(2–42)_ = 535.298, *p* < 0.001) with the increase, both in TPH2^+/+^ and TPH2^−/−^, from pnd10 to pnd30 (TPH2^+/+^/pnd30: +2105%, *p* < 0.001 vs. TPH2^+/+^/pnd10; TPH2^−/−^/pnd30: +5705%, *p* < 0.001 vs. TPH2^−/−^/pnd10; Figure 2H).

### Analysis of Stress Responsiveness in TPH2^−/−^ Male and Female Adult Rats

In order to evaluate if the lack of serotonin influences the response to an acute stress challenge, we exposed the animals to 1 h of restraint stress and sacrificed them immediately at the end of the stress session (stress0’) or 1 h later (stress60’).

**Figure 3:**
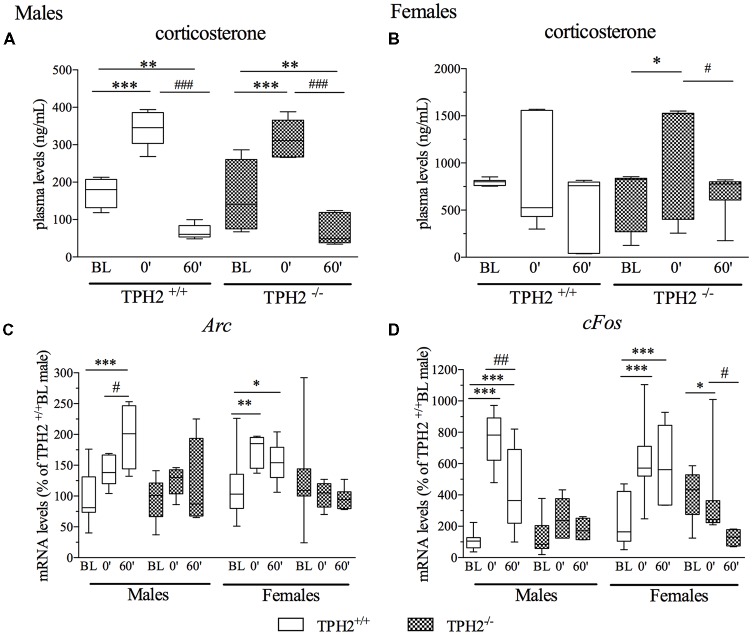
Analysis of corticosterone plasma levels **(A,B)** and activity regulated cytoskeleton associated protein (*Arc*: **C**) and Fos Proto-Oncogene (*cFos*: **D**) mRNA levels in the PFC of TPH2^+/+^ and TPH2^−/−^ adult male and female rats, exposed to an acute restraint stress. The corticosterone data represent the mean ± SEM of at least four independent determinations. Gene expression data are expressed as mean % of TPH2^+/+^/baseline (BL) males ± SEM of at least four independent determinations. *p < 0.05, **p < 0.01, ***p < 0.001 vs. BL/same genotype; ^#^*p* < 0.05, ^##^*p* < 0.01; ^###^*p* < 0.001 vs. stress0’/same genotype (two-way ANOVA with Fisher’s PLSD panel **A,B**; three-way ANOVA with Fisher’s PLSD panel **C,D**).

In male rats, plasma corticosterone levels were significantly modulated by the acute stress (*F*_(1–27)_ = 60.729, *p* < 0.001). Indeed, we found a strong increase in corticosterone levels in rats of both genotypes at stress0’ time point in comparison to BL conditions (unstressed rats; TPH2^+/+^/stress0’: +99%, *p* < 0.001 vs. TPH2^+/+^/BL; TPH2^−/−^/stress0’: +98%, *p* < 0.001 vs. TPH2^−/−^/BL), while a significant decrease was observed in the group of rats killed at stress60’ time point compared to BL groups of the same genotype (TPH2^+/+^/stress60’: −61%, *p* < 0.01 vs. TPH2^+/+^/BL; TPH2^−/−^/stress60’: −54%, *p* < 0.05 vs. TPH2^−/−^/BL) as well as to stress0’ groups (TPH2^+/+^/stress60’: −81%, *p* < 0.001 vs. TPH2^+/+^/stress0’; TPH2^−/−^/stress60’: −77%, *p* < 0.001 vs. TPH2^−/−^/stress0’; Figure 3A).

As shown in Figure 3B, female rats showed high heterogeneity in corticosterone levels at BL and in response to stress. Statistical analysis revealed significant modulation of plasma corticosterone levels by acute stress only in TPH2^−/−^ rats (*F*_(1–42)_ = 5.239, *p* < 0.05), with an upregulation of corticosterone levels in stress0’ group compared to BL (+82%, *p* < 0.05 vs. TPH2^−/−^/BL) and a decrease at stress60’ time point with respect to TPH2^−/−^/stress0’ (−50%, *p* < 0.05 vs. TPH2^−/−^/stress0’).

### Analysis of the Immediate Early Genes *Arc* and *cFos* Following Stress

Since we previously demonstrated the implication of the immediate early genes in the response to acute stress (Molteni et al., 2008) and their modulation due to the deletion of the SERT (Molteni et al., 2010), here, we measured two markers of neuronal activation, *Arc* and *cFos*, in order to evaluate if the lack of serotonin in the CNS influenced the response to the restraint stress.

As shown in Figure 3C, three-way ANOVA did not reveal a significant genotype × stress × sex interaction (*F*_(2–101)_ = 1.668, *p* > 0.05) on the expression of *Arc*. Nevertheless, we found a significant effect of the stress (*F*_(2–101)_ = 5.692, *p* < 0.01) with a genotype × stress interaction (*F*_(2–101)_ = 7.181, *p* < 0.01). Accordingly, *Arc* mRNA levels were increased by stress exposure only in the TPH2^+/+^ rats of both sexes (TPH2^+/+^/stress60’/male: +97%, *p* < 0.001 vs. TPH2^+/+^/BL/male; TPH2^+/+^/stress60’/male: +38%, *p* < 0.05 vs. TPH2^+/+^/stress0’/male; TPH2^+/+^/stress0’/female: +54%, *p* < 0.01 vs. TPH2^+/+^/BL/female; TPH2^+/+^/stress60’/female: +42%, *p* < 0.01 vs. TPH2^+/+^/BL/female). Interestingly, this stress-mediated upregulation of *Arc* expression was completely blunted in TPH2^−/−^ rats.

Similarly, *cFos* expression was not significantly modulated by genotype × stress × sex interaction (*F*_(2–90)_ = 2.170, *p* > 0.05). However, a significant effect of the stress (*F*_(2–90)_ = 20.596, *p* < 0.001) with a genotype × stress interaction (*F*_(2–90)_ = 19.110, *p* < 0.001) was observed. *Post hoc* analysis revealed a strong increase in *cFos* mRNA levels in TPH2^+/+^ male rats at both time points after stress in comparison to BL (TPH2^+/+^/stress0’/male: +662%, *p* < 0.001 vs. TPH2^+/+^/BL/male; TPH2^+/+^/stress60’/male: +336%, *p* < 0.01 vs. TPH2^+/+^/BL/male). The upregulation of the *cFos* mRNA levels peaked at stress0’ was markedly attenuated 1 h later (−43%, *p* < 0.01 vs. TPH2^+/+^/stress0’/male), confirming previously published data (Durchdewald et al., 2009). In female, acute challenge induced an upregulation of *cFos* mRNA levels in the TPH2^+/+^/stress0’ rats (+160%, *p* < 0.001 vs. TPH2^+/+^/BL/female) and stress60’ group (+145%, *p* < 0.01 vs. TPH2^+/+^/BL/female). This characteristic pattern was again not observed in TPH2^−/−^ female rats; moreover, 1 h after the stress exposure we found a significant downregulation of *cFos* mRNA levels in TPH2^−/−^ rats (TPH2^−/−^/stress60’/female: −67%, *p* < 0.01 vs. TPH2^−/−^/BL/female; TPH2^−/−^/stress60’/female: −65%, *p* < 0.01 vs. TPH2^−/−^/ stress0’/female; Figure 3D).

### Analysis of *Bdnf* Transcripts Following Stress

**Figure 4:**
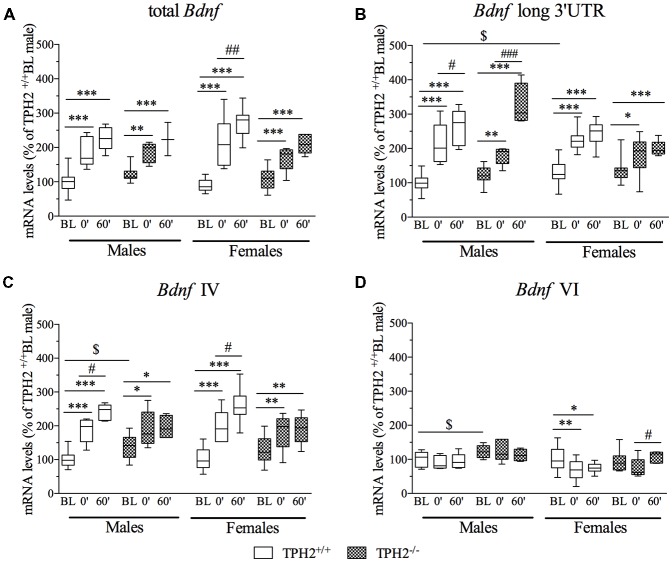
Analysis of total *Bdnf*, **(A)** *Bdnf* long 3’UTR, **(B)** *Bdnf* isoform IV and **(C)** *Bdnf* isoform VI **(D)** mRNA levels in the PFC of TPH2^+/+^ and TPH2^−/−^ adult male and female rats, exposed to an acute restraint stress. The data, expressed as % of TPH2^+/+^/BL males, represent the mean ± SEM of at least four independent determinations. *p < 0.05, **p < 0.01, ***p < 0.001 vs. BL/same genotype; ^#^*p* < 0.05, ^##^*p* < 0.01; ^###^*p* < 0.001 vs. stress0’/same genotype, ^$^*p* < 0.05 vs. TPH2^+/+^ /BL/male (three-way ANOVA with Fisher’s PLSD).

The three-way ANOVA revealed no effect of the interaction genotype × stress × sex (*F*_(2–99)_ = 1.754, *p* > 0.05) on total *Bdnf* gene expression (Figure 4A). However, we found a significant effect of stress (*F*_(2–99)_ = 112.032, *p* < 0.001). Accordingly, total *Bdnf* was increased in both TPH2^+/+^ and TPH2^−/−^ male rats exposed to the acute stress and sacrificed at time point stress0’ (TPH2^+/+^/Stress0’: +87%, *p* < 0.001 vs. TPH2^+/+^/BL; TPH2^−/−^/Stress0’: +51%, *p* < 0.01 vs. TPH2^−/−^/BL) and at time 60’ (TPH2^+/+^/stress60’: +127%, *p* < 0.001 vs. TPH2^+/+^/BL; TPH2^−/−^/Stress60’: +82%, *p* < 0.001 vs. TPH2^−/−^/BL).

In the female rats, the exposure to stress increased the expression of total *Bdnf*, at both time points, in TPH2^+/+^ (TPH2^+/+^/stress0’: +146%, *p* < 0.001 vs. TPH2^+/+^/BL; TPH2^+/+^/stress60’: +203%, *p* < 0.001 vs. TPH2^+/+^/BL) as well as in TPH2^−/−^ (TPH2^−/−^/stress0’: +51%, *p* < 0.01 vs. TPH2^−/−^/BL; TPH2^−/−^/stress60’: +88%, *p* < 0.001 vs. TPH2^−/−^/BL). Moreover, TPH2^+/+^stressed rats sacrificed 1 h after the stress exposure had higher mRNA levels compared to the stress0’ groups (TPH2^+/+^/stress60’: +23%, *p* < 0.01 vs. TPH2^+/+^/stress0’).

Furthermore, the significant genotype × stress interaction (*F*_(2–98)_ = 6.490, *p* < 0.01) indicates that the upregulation found in TPH2^+/+^ was higher than the one observed in TPH2^−/−^ rats.

*Bdnf* long 3’UTR mRNA levels (Figure 4B) were significantly modulated by the interaction genotype × stress × sex (*F*_(2–101)_ = 3.203, *p* < 0.05). In particular, we found a significant genotype × sex interaction (*F*_(2–101)_ = 7.205, *p* < 0.01) reflecting an upregulation of *Bdnf* long 3’UTR expression in female TPH2^+/+^/BL compared to the male counterpart (TPH2^+/+^/BL: +30%, *p* < 0.05 vs. TPH2^+/+^/BL). Moreover, the significant effect of stress (*F*_(2–101)_ = 109.926, *p* < 0.001) indicated that *Bdnf* long 3’UTR was increased after the acute challenge in male (TPH2^+/+^/stress0’/male: +112%, *p* < 0.001 vs. TPH2^+/+^/BL/male; TPH2^+/+^/stress60’/male: +161%, *p* < 0.001 vs. TPH2^+/+^/BL/male; TPH2^−/−^/stress0’/male: +46%, *p* < 0.01 vs. TPH2^−/−^/BL/male; TPH2^−/−^/stress60’/male: +165%, *p* < 0.001 vs. TPH2^−/−^/BL/male) and in female (TPH2^+/+^/stress0’/female: +73%, *p* < 0.001 vs. TPH2^+/+^/BL/female; TPH2^+/+^/stress60’/female: +88%, *p* < 0.001 vs. TPH2^+/+^/BL/female; TPH2^−/−^/stress0’/female: +31%, *p* < 0.05 vs. TPH2^−/−^/BL/female; TPH2^−/−^/stress60’/female: +46%, *p* < 0.001 vs. TPH2^−/−^/BL/female), independently from the genotype.

Moreover, in male rats the up-regulation observed 1 h after the stress exposure was significantly higher than the effect found at time 0’ in both genotype (TPH2^+/+^/stress60’/male: +23%, *p* < 0.001 vs. TPH2^+/+^/stress0’/male; TPH2^−/−^/stress60’/male: 81%, *p* < 0.001 vs. TPH2^−/−^/stress0’/male).

Finally, the significant genotype × stress interaction (*F*_(2–101)_ = 4,368, *p* < 0.05) indicates that the increase in expression of *Bdnf* long 3’UTR due to stress exposure was more robust in TPH2^+/+^ than in TPH2^−/−^ rats.

Analysis of *Bdnf* isoform IV mRNA levels (Figure 4C) revealed no effect of the genotype × stress × sex interaction (*F*_(2–101)_ = 0.094, *p* > 0.05), but a significant effect of stress (*F*_(2–101)_ = 66.292, *p* < 0.001). In male, *Bdnf* isoform IV expression was upregulated by stress in TPH2^+/+^ rats (TPH2^+/+^/stress0’/male: +88%, *p* < 0.001 vs. TPH2^+/+^/BL/male; TPH2^+/+^/stress60’/male: +141%, *p* < 0.001 vs. TPH2^+/+^/BL/male) with a further increase from stress point 0’ to 60’ (TPH2^+/+^/stress60’/male: +28%, *p* < 0.05 vs. TPH2^+/+^/stress0’/male) and in TPH2^−/−^ (TPH2^−/−^/stress0’/male: +37%, *p* < 0.01 vs. TPH2^−/−^/BL/male; TPH2^−/−^/stress60”/male: +41%, *p* < 0.01 vs. TPH2^−/−^/BL/male). Moreover, *Bdnf* IV was increased in unstressed TPH2^−/−^ in comparison to TPH2^+/+^ rats (TPH2^−/−^/BL/male: +39%, *p* < 0.01 vs. TPH2^+/+^/BL/male).

In female, exposure to the acute stress increased the *Bdnf* isoform IV mRNA levels in both genotypes (TPH2^+/+^/stress0’/female: +98%, *p* < 0.001 vs. TPH2^+/+^/BL/female; TPH2^+/+^/stress60’/female: +152%, *p* < 0.001 vs. TPH2^+/+^/BL/female; TPH2^+/+^/stress60’/female: +27%, *p* < 0.05 vs. TPH2^+/+^/stress0’/female; TPH2^−/−^/stress0’/female: +39%, *p* < 0.05 vs. TPH2^−/−^/BL/female; TPH2^−/−^/stress60’/female: +46%, *p* < 0.01 vs. TPH2^−/−^/BL/female). Similar to total *Bdnf* and *Bdnf* long 3’UTR, the significant genotype × stress interaction (*F*_(2–101)_ = 11.065, *p* < 0.001) was also observed for the *Bdnf* isoform IV, suggesting a different response to the challenging situation in the two genotypes in terms of magnitude of the effect.

Similarly, *Bdnf* isoform VI mRNA levels (Figure 4D) were not affected by the genotype × stress × sex interaction (*F*_(2–100)_ = 1.741, *p* > 0.05). However, the significant effect of the genotype (*F*_(2–100)_ = 10.338, *p* < 0.01) supports the slightly higher mRNA levels found in unstressed male TPH2^−/−^ compared to unstressed TPH2^+/+^ male rats (TPH2^−/−^/BL/male: +22%, *p* < 0.05 vs. TPH2^+/+^/BL/male).

Moreover, in females, isoform VI expression was downregulated specifically in TPH2^+/+^ rats (TPH2^+/+^/stress0’/female: −32%, *p* < 0.05 vs. TPH2^+/+^/BL/female; TPH2^+/+^/stress60’/female: −26%, *p* < 0.05 vs. TPH2^+/+^/BL/female), while its levels were increased in TPH2^−/−^ stressed rats sacrificed 1 h after the stress exposure in comparison to the animals of the same genotype killed immediately after the acute stress (TPH2^+/+^/stress60’/female: +45%, *p* < 0.05 vs. TPH2^−/−^/stress0’/female).

## Discussion

The results of the present study highlight that the deletion of *Tph2* profoundly affects neuroplastic mechanisms from the first stage of life until adulthood and influences the response to an acute stress.

We found a significant increase of total *Bdnf* mRNA levels in both adult TPH2^−/−^ male and female rats. Interestingly, the contribution of different *Bdnf* isoforms to this increase is sex-specific: in male TPH2^−/−^ rats, it is due to enhancement of all *Bdnf* transcripts investigated, while in females only the isoform IV is upregulated.

These results are in line with our previous data obtained in a rat model with increased extracellular 5-HT levels, SERT-deficient rats (Molteni et al., 2010; Calabrese et al., 2013). While hyperserotonergia induced by SERT-deletion (Homberg et al., 2007; Olivier et al., 2008) impairs neuronal plasticity in the PFC and hippocampus by downregulation of *Bdnf*, serotonin depletion in the adult CNS has an opposite effect, confirming the strict dependence of *Bdnf* expression on serotonergic status.

These effects were mirrored, at translational level, by an upregulation of the mature form of BDNF protein levels in the crude synaptosomal fraction in both male and female TPH2^−/−^ rats. The enhancement of the protective form of the neurotrophin, that regulates synaptic connections and plasticity (Martinowich et al., 2007) may be indicative for an increase in the pool of the neurotrophin ready for the release (Poo, 2001; Lau et al., 2010). This suggests a potential compensatory mechanism in the brain to deal with the absence of the trophic contribution of serotonin, confirming data previously obtained in PFC (Kronenberg et al., 2016) and hippocampus (Migliarini et al., 2013) of TPH2-deficient mice.

Furthermore, it’s well known that ontogenesis is a critical period for brain adaptability and plasticity. Several lines of evidence support the fundamental role of 5-HT during neurodevelopment (Lauder, 1993; Gaspar et al., 2003) as well as that BDNF is a crucial molecule in promoting CNS growth and in the establishment of neural circuitry including the serotoninergic system (Mamounas et al., 1995). Accordingly, we observed a stepwise increase in *Bdnf* expression from pnd1 to pnd30 in wild type animals as previously demonstrated (Calabrese et al., 2013). Although a similar profile was observed in TPH2^−/−^ rats, the comparison between the two genotypes revealed a significant increase in total *Bdnf* mRNA levels in both male and female TPH2^−/−^ rats at pnd30. In males, this effect was paralleled by an upregulation of *Bdnf* isoform IV and VI, while in females this effect was, again, restricted to the specific effect on the expression of the isoform IV. However, no differences between genotypes at earlier time points (at birth and at pnd10), or even downregulation in *Bdnf* expression levels in TPH2^−/−^ in comparison to TPH2^+/+^ pnd10 male rats were observed, suggesting that peripheral sources may compensate for the lack of central 5-HT synthesis during the early postnatal periods of life. Indeed, the blood brain barrier is not fully functional before pnd12 (Ribatti et al., 2006) and 5-HT from the placenta at embryonic stages (Cool et al., 1990; Carrasco et al., 2000) and from the peripheral blood after birth may easily enter the brain. Accordingly, Vitalis et al. (2007) demonstrated that the embryonic transient 5-HT depletion did not modify cortical BDNF levels until PND21 in pups. In summary, these results suggest that the activation of trophic mechanisms set in motion to compensate the serotonin synthesis deficiency in the CNS became more evident starting from pnd30, when there is no more supply of 5-HT from alternative sources, such as placenta and peripheral blood.

Moreover, the different regulation of *Bdnf* isoforms we found in females in comparison to males may be due to the fact that different transcripts may have different subcellular localization (Chiaruttini et al., 2008) and are controlled by specific intracellular pathways (Aid et al., 2007).

Among these, sex hormones are known to contribute to BDNF modulations (Chan and Ye, 2017), in line with the finding that estrogens can induce *Bdnf* expression by activating their receptors and may modify the neurotrophin activity through methylation of the *Bdnf* promoter IV and V in the hippocampus (Moreno-Piovano et al., 2014). Moreover, sex steroids influence serotonergic neurotransmission (Dalla et al., 2005; Kokras et al., 2009). Indeed, for example it has been demonstrated that androgens facilitate the 5-HT binding to its transporter SERT, while estrogens seem to delay it (Kranz et al., 2015). Due to the limited information available on the functional role of each *Bdnf* transcript, it is not feasible to draw clear-cut conclusions on the consequences exerted by differences in isoform expression between male and female.

Seeing that, in basal condition, 5-HT deficiency was in some way compensated by neuroplastic mechanisms and considering the well-established relationship between the serotonergic system and the stress reactivity (Chen and Miller, 2012; Homberg et al., 2014) we then tested if the lack of central serotonin affects the ability to cope with more dynamic conditions. So, we exposed adult rats to 1 h of restraint stress and we sacrificed them immediately at the end of the stress session or 1 h later. We observed that corticosterone release was differently modulated by stress in wild type males and females and it was affected by lack of 5-HT in a sex specific manner. Indeed, as expected from the literature (Adzic et al., 2009; Molteni et al., 2009) stress exposure induced a strong release of corticosterone immediately after the restraint stress in TPH2^+/+^ male rats and a significant decrease 1 h later, while in females this pattern was not so pronounced and was statistically significant only in TPH2^−/−^, but not in TPH2^+/+^ rats. Corticosterone levels in females are highly influenced by the estrus cycle (Atkinson and Waddell, 1997) and by the stress (Figueiredo et al., 2002). Since females used in our study were not synchronized, high variability in the corticosterone levels in female rats of both genotypes might originate from different estrus cycle stages.

Furthermore, the implication of neuronal activation in response to acute stress can be evaluated by the expression of immediate early genes (Ons et al., 2004; Molteni et al., 2008), in order to assess a different outcome to the acute restraint stress exposure driven by 5-HT deficiency, we measured two markers of neuronal activation, *Arc* and *cFos*. As expected, in both males and female wild type rats we observed a clear stress response, with the slow increase in *Arc* expression during and 1 h after stress and quick upregulation at stress0 and downregulation 1 h later in *cFos* expression, confirming previously published data (Durchdewald et al., 2009). Interestingly in both males and females, the lack of 5-HT in the CNS influenced the response to the challenging condition by preventing the upregulation of both the immediate early genes mRNA levels found in TPH2^+/+^ rats. Seen that Arc mediates the translation between neuronal activation-induced changes into sustained structural and functional modification at synaptic levels (Alberi et al., 2011), the lack of activation observed in TPH2^−/−^ rats may reflect an incorrect translation of the stimulus into a more stable outcome.

Moreover, we have previously shown that hyperserotonergia, evoked by the lack of SERT, has an opposite effect on the stress-induced neuronal activation, leading to a more pronounced upregulation of *Arc* in SERT-deficient rats in comparison to wild types (Molteni et al., 2008). So, these opposite responses in the neuronal activation of PFC induced by acute stress in hyper- (SERT-deficient) and hyposerotonergic (TPH2-deficient) rats further support an important role of 5-HT in stress response. Accordingly, it was recently shown that the 5-HT_2_ receptor antagonist ketanserin also blocks *Arc* induction in the PFC in response to a stress paradigm (Benekareddy et al., 2011). Remarkably, this serotonin effect on neuronal activation seems to be completely independent from the classical stress response via the hypothalamus-pituitary adrenal axis which is hardly affected by the absence of serotonin.

Finally, we found that, despite the increased levels of BDNF observed in TPH2^−/−^, rats, this compensatory mechanism was not enough to restore the normal ability to react to the acute stress challenge, probably because of the complexity of the system and circuit involved that may be BDNF-independent. Even if *Bdnf* transcripts were upregulated in both genotypes in response to stress, the effects found in TPH2^+/+^ were stronger than those induced in the TPH2^−/−^ rats. The fact that BDNF is upregulated by stress in the PFC even in the absence of serotonin, while the immediate early genes are not, suggests distinct stress-modulated neural circuits whereby one is serotonergic, and another is 5-HT independent.

Taken together our findings suggest that 5-HT deficiency in the brain modulates neuroplasticity probably to compensate for the lack of trophic support provided by serotonin starting after weaning until adulthood. Nevertheless, in response to a stressful condition the system is not able to properly respond by setting in motion the strategies to cope with an acute challenge.

## Author Contributions

FC and NA: conception and study design. PB, PP, MT and GS performed experiments. PB, PP, FC and NA performed data analysis and interpretation of the data. PB drafted and FC, NA and MB critically revised the manuscript. All authors critically reviewed the content and approved the final version for publication.

## Conflict of Interest Statement

The authors declare that the research was conducted in the absence of any commercial or financial relationships that could be construed as a potential conflict of interest.

## Abbreviations

5-HT: 5-hydroxytryptamine
*Arc*: activity regulated cytoskeleton associated protein
ANOVA: analysis of variance
BL: baseline
BDNF: brain-derived neurotrophic factor
CNS: central nervous systems
PLSD: Fisher’s Protected Least Significant Difference
*cFOS*: Fos Proto-Oncogene
mBDNF: mature BDNF
pnd: postnatal day
PFC: prefrontal cortex
RT-PCR: real-time polymerase chain reaction
SERT: serotonin transporter
SEM: standard error mean
TPH2: tryptophan hydroxylase 2.
